# Graph Curvature for Differentiating Cancer Networks

**DOI:** 10.1038/srep12323

**Published:** 2015-07-14

**Authors:** Romeil Sandhu, Tryphon Georgiou, Ed Reznik, Liangjia Zhu, Ivan Kolesov, Yasin Senbabaoglu, Allen Tannenbaum

**Affiliations:** 1 Departments of Computer Science and Applied Mathematics/Statistics, Stony Brook University, Stony Brook, NY 11794; 2 Department of Electrical and Computer Engineering, University of Minnesota, Minneapolis, MN 55455; 3 Computational Biology, Memorial Sloan Kettering, New York, NY 10065.

## Abstract

Cellular interactions can be modeled as complex dynamical systems represented by weighted graphs. The functionality of such networks, including measures of robustness, reliability, performance, and efficiency, are intrinsically tied to the topology and geometry of the underlying graph. Utilizing recently proposed geometric notions of curvature on weighted graphs, we investigate the features of gene co-expression networks derived from large-scale genomic studies of cancer. We find that the curvature of these networks reliably distinguishes between cancer and normal samples, with cancer networks exhibiting higher curvature than their normal counterparts. We establish a quantitative relationship between our findings and prior investigations of network entropy. Furthermore, we demonstrate how our approach yields additional, non-trivial pair-wise (i.e. gene-gene) interactions which may be disrupted in cancer samples. The mathematical formulation of our approach yields an exact solution to calculating pair-wise changes in curvature which was computationally infeasible using prior methods. As such, our findings lay the foundation for an analytical approach to studying complex biological networks.

The onset and proliferation of cancer stems from dynamic changes that result from a series of changes in cellular interactions governing a complex network^1^,^2^,^3^. Inspired by the previous work of Teschendorff^4^, examining properties of such networks at various states may aid in the understanding of certain cellular processes leading to tumorigenesis. One key property is the notion of robustness, or the ability of a system to adapt to dynamic changes and perturbations while still maintaining functionality. From this perspective, a fundamental hurdle to cancer therapy is acquired tumor robustness^5^. On the other hand, quantification of robustness and in particular, that of cancer networks, has remained elusive. Understanding and exploiting such network properties from a biological perspective provides an alternative framework to viewing underlying mechanisms. In turn, this may guide and uncover new drug targets.

In this work, we demonstrate the role of curvature as system-level characteristic of certain cancer networks and its relationship to network functionality in terms of a notion of robustness^6^,^7^ , specifically at the local interaction level. Curvature, in the broad sense, is a measure by which a geometrical object deviates from being flat and is defined in varying manners given the context^8^. Our reference to curvature will be restricted to **Ricci curvature**** and its contraction, ***scalar curvature***. Similarly, “robustness” can be formally defined in terms of the rate function from the theory of large deviations by appealing to the Fluctuation Theorem^6^,^7^. Roughly speaking, robustness relates to the rate at which a given dynamical system returns to its original (normal) state following a perturbation or external disturbance. The key ingredient that intimately links curvature and robustness is the concept of entropy. Indeed, through a suitable characterization on the lower bound of Ricci curvature^9^, one can show that entropy and curvature are positively correlated, a fact that we express as Δ*S* × Δ*Ric* ≥ 0 and where Δ*S* and Δ*Ric* are the changes in entropy and Ricci curvature, respectively (see Methods)^10^. Now, if we consider random perturbations to the network, the Fluctuation Theorem asserts that Δ*S* × Δ*R* ≥ 0 where Δ*R* is a relative change in robustness and hence, the relationship Δ*Ric* × Δ*R* ≥ 0 holds. As we will argue in this work, this tacit relationship to robustness will allow curvature to serve as an alternative, yet powerful proxy (Fig. 1). This seems especially true for cancer networks.

Our work here differs from previous approaches of characterizing network robustness^4^,^6^,^7^,^11^ in several important aspects. To the best our knowledge, it is the first to express general network functional robustness through curvature and to point out that this may provide an intrinsic cancer characteristic. With regards to entropy, our utilization of curvature holds the following key advantages: (I) Ricci curvature provides *pairwise information* over all possible pathways as opposed to network entropy, which is defined as a nodal measure^7^. This is particularly significant due to the fact that we are interested in specific gene-to-gene interactions contributing to the resilience of cancer (including those “hidden” interactions not necessarily defined by the underlying topology). In short, previous work of network entropy exhibits a “loss of information” with regards to the robustness of the interactions themselves^4^,^7^. (II) Ricci curvature can be formulated as a simple linear program and is well-behaved as compared to network entropy^12^. (III) Scalar curvature, in a similar manner to network entropy, is defined as a nodal measure in which interactions are not explicitly described.

In the present work, we compare gene co-expression networks from cancer and adjacent-normal tissue samples using network curvature. Motivated by previous entropic studies^4^, we fix the underlying topology of the networks using prior data on known physical interactions between gene products allowing only the weights to evolve between normal and tumor networks. Then, by treating each network as a random walk, we attempt to exploit the underlying dynamics of specific gene-to-gene interactions.

Finally, we should note that the methods explicated in the present work are applicable not only to cancer networks, but may also assist in unifying several phenomena in molecular biology for which notions of robustness (via entropy and curvature) seem to be increasingly important^5^,^13^,^14^,^15^. For example, recent work has demonstrated that local signaling entropy may serve as a novel indicator of drug sensitivity^13^ while at the same time, may operate as a proxy for the height or elevation in Waddington’s differentiation landscape^14^. Furthermore, it has been argued that feedback loops are essential to the function of biological mechanisms and systems that arise from deliberate Darwinian-like principles^5^,^15^. In what follows, one can view Ricci curvature as a new feedback measure, i.e., the number of triangles in a network (redundant pathways) can be characterized by a lower bound of Ricci curvature^5^,^16^,^17^. This fascinating interplay between feedback, robustness, entropy, and now Ricci curvature is at the core of this work.

The remainder of this paper is outlined as follows. We first provide results to demonstrate that Ricci curvature, more precisely Ollivier-Ricci curvature^18^,^19^, is a proxy for robustness as well as an apparent cancer characteristic. In particular, we discuss the importance (and previously unresolved) ability of quantifying robustness at the interaction level. We then show that several analogous nodal curvature measures, defined through varying contractions of Ricci curvature, achieve similar results to that of network entropy, which by construction, is a nodal attribute. We conclude with a discussion of the results with a primary focus on information loss from previous entropic methods, examination of robustness for specific gene-to-gene interactions in context of cancer biology, and analytic advantages of employing Ricci curvature as opposed to entropy. From this, we then offer a possible path forward that relates the well-known Ricci flow to the effect (and design thereof) of specific drug targets that can possibly mitigate the robust nature of cancer.

## Results

We focus our investigation primarily on transcription networks composed of metabolic and cancer specific genes^20^,^21^. For each data set, gene co-expression networks were generated by calculating the non-parametric (Spearman) correlation between all pairs of genes. That is, for a given gene pair, correlation was computed across all samples within a given phenotype (normal or cancerous tissue). The metabolic data set consists of approximately 1600 metabolic genes (derived from the Recon2 human metabolic reconstruction^22^) of six different tumor types: breast cancer (BRCA^M^), head and neck squamous cell carcinoma (HNSC^M^), kidney papillary carcinoma (KIRP^M^), liver cancer (LIHC^M^), lung adenocarcinoma (LUAD^M^), and thyroid cancer (THCA^M^). We further supplemented the above study with corresponding networks that contain approximately 500 *cancer-related* genes derived from the Cosmic Cancer Gene Census^21^ (denoted by ^T^, e.g., BRCA^T^). With regards to the topology, the networks analyzing metabolic genes possess a total of 33843 edges, average degree of 43, and a median degree of 34. For the networks composed of known cancer-related genes, the total number of edges, average degree, and median degree are 8162, 37, and 22 respectively (see Methods).

### Gene-to-Gene Robustness: Ollivier-Ricci Curvature

**Figure 2:**
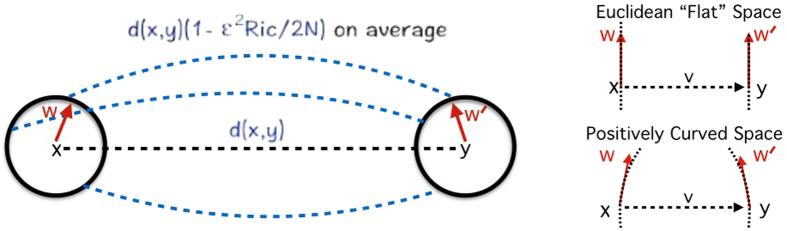
Positive Ricci curvature is reflected by the characteristic that for two very close points *x* and *y* with a tangent vector *v* connecting *xy* as well as tangent vectors *w* (at *x*) and *w*′ (at *y*), in which *w*′ is obtained by parallel transport of *w*, that the two corresponding geodesics will get closer. This can be compared to the traditional flat geometry of a Euclidean space where such distances are unaffected during the parallel transport. Equivalently, this may be formulated by the fact that the transportation distance between two small (geodesic balls) is less than the distance of their centers. Ricci curvature along the direction *xy* quantifies this, averaged on all directions *w* at *x*.

**Figure 3:**
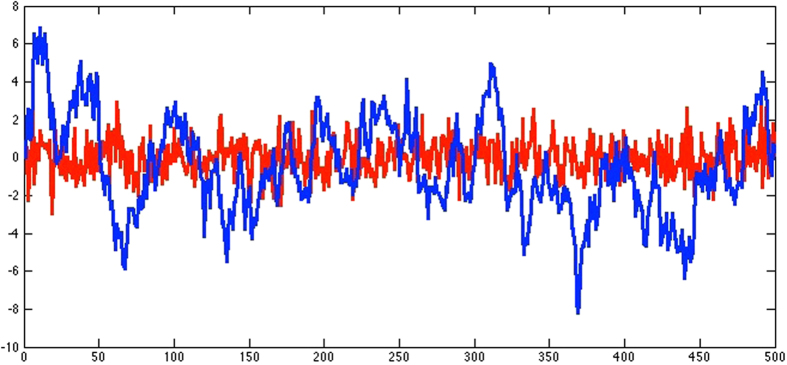
We generated two Ornstein-Uhlenbeck processes with the same parameter set except for different *α* and in turn, exhibits different Ollivier-Ricci curvatures: *κ*(*x*, *y*) = 0.6321 (red) with *α* = 1.0 and *κ*(*x*, *y*) = 0.0952 with *α* = 0.1 For both signals, Ornstein-Uhlenbeck process parameters were initialized with x(0) = 1 with *σ* = 1. One can see that Δ*κ* × Δ*α* ≥ 0 for this broad set of stochastic processes.

**Figure 4:**
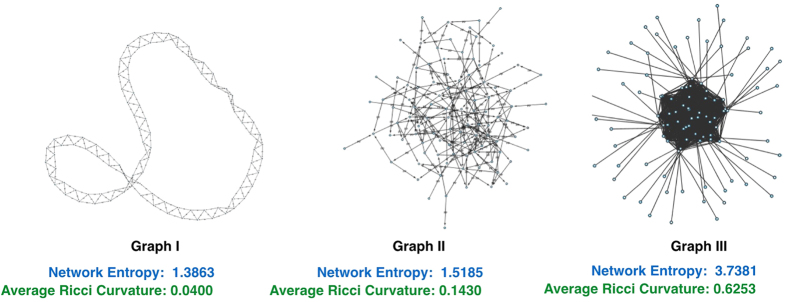
We computed the average Ollivier-Ricci curvature for three different networks shown above as well as network entropy. To ensure a fair comparison, each of the networks is composed of 200 nodes with 400 (unweighted) edges - the only difference in the underlying structure. Although Ricci curvature is a local property, it nevertheless shows that, on average, Ricci curvature is higher for networks that exhibit higher entropy.

We employ a neat notion of a Ricci curvature^19^ inspired through coarse geometry (Fig. 2). In particular, if we let (*X*, *d*) be a metric space equipped with a family of probability measures {*μ*_*x*_:*x* ∈ *X*}, we define the *Ollivier-Ricci curvature κ*(*x*, *y*) along the geodesic connecting nodes *x* and *y* via


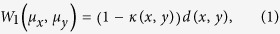


where *W*_1_ denotes the Earth Mover’s Distance (Wasserstein 1-metric)^23^,^24^, and *d* is the geodesic distance on the graph. For the case of weighted graphs, we set


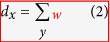



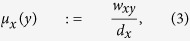


where *d*_*x*_ is the sum taken over all neighbors of node *x* and where *w*_*xy*_ denotes the weight of an edge connecting node *x* and node *y* (*w*_*xy*_ = 0 if *d*(*x*, *y*) ≥ 2). The measure *μ*_*x*_ may be regarded as the distribution of a one-step random walk starting from *x*, with the weight *w*_*xy*_ quantifying the strength of interaction between nodal components or the diffusivity across the corresponding link (edge). To motivate this definition and highlight the role of curvature as a proxy for robustness, we compute the Ollivier-Ricci curvature for two Ornstein-Uhlenbeck^19^ processes generated in an identical manner except with two different “mean-reversion” rates (see Methods). An Ornstein-Uhlenbeck process describes velocity of a Brownian particle (with mass) under the influence of friction, and is regarded as more realistic than simple Brownian motion. In particular, this illustrative example (Fig. 3) shows that the signal with higher curvature (red) is more capable of returning towards zero (equilibrium) in the face of the same noise (perturbations), illuminating its robustness and as argued previously via the Fluctuation Theorem. One may also consider, for motivational purposes, Ollivier-Ricci curvature on several networks with differing geometries and topologies, and their functionality with respect to robustness (Fig. 4, Fig. S1). Nevertheless, the positive correlation between the rate of return to equilibrium in the Ornstein-Uhlenbeck sense and Ollivier-Ricci curvature, holds for higher dimensions and provides a simple yet informative example linking curvature to robustness.

We then compute the Ollivier-Ricci curvature on tumor and normal tissue networks for all the studied cancer types. We begin with a characterization of the distributions for all networks composed of metabolic genes (≈1.25 M possible pairs) as well as our supplemental corresponding networks for which we examine only known cancer related genes (≈100 K possible pairs). In particular, we provide an analysis in terms of average curvature, the difference in expected value on upper/lower 5% tails of distribution along with the p-value result of a paired one-tailed Wilcoxon signed-rank test^25^ (Table 1, Table S1). This analysis is done, in part, to characterize the shift of distribution with respect to (cancer-normal) changes in Ollivier-Ricci curvature. As such, one can see that the difference between cancer and normal tissue distributions is “positive” with a low p-value signifying robustness. Further, one can consider the left tail of the distribution (at a given 0.1% 0.5%, 1%, 3%) as the lower bound of Ollivier-Ricci curvature as opposed to simply taking minimum value which is sensitive to topological errors. Then, it can be seen that this increase in lower bound points precisely to an increase in entropy^9^ (Table S2, Table S3). In all cases of examining the left tail (12 cases at 5 given lengths), the lower bound for a particular cancer network was larger than its normal counterpart. The trend also became more apparent as we decreased the tail length. The largest tail length of 5% was chosen as this was in line with the Wilcoxon signed-rank test. We also note that while we do not restrict our computation to node degree or path length, i.e., curvature is assigned to every gene pair, the average statistic was taken over those interactions with *d*(*x*, *y*) = 1. Revisiting equation (1), one can see curvature (and changes in curvature) for interactions “far” from the underlying topology will decay due to the term *d*(*x*, *y*). We should note that since a graph is a 1-geodesic space, if *κ*(*x*, *y*) ≥ *k* for *d*(*x*, *y*) = 1, then *κ*(*x*, *y*) ≥ *k* " *x*, *y*^18^. Thus, computing statistics (i.e., averages) for adjacent vertices will suffice and results are still valid (in the sense of robustness) for *d*(*x*, *y*) ≥ 2, e.g., non-adjacent pairs in general will contribute negligibly and can be treated as scaling such statistics.

We note that the primary advantage of employing Ollivier-Ricci curvature is its ability to characterize robustness at the interaction level (as opposed to genes where entropic measures are just defined at the nodal level). In particular, we first report the top and bottom ten interactions with respect to changes in Ollivier-Ricci curvature for the case of BRCA^T^ (Table 2, Table 3). The investigation of this network is particularly compelling as we sought to find a subset of *interactions* that contribute to the network resilience (and/or fragility) amongst a set of known cancer related genes. We observe the gene RNF43 exhibits several robust and fragile pathways: RNF43-RSOP3, RNF43-RSOP2, RNF43-TP53, RNF43-NONO, RNF43-POT1. This is a surprising result given that RNF43 physically interacts with very few gene products and in general, is associated as a tumor suppressor in ovarian cancer^26^. On the other hand, RNF43 dominates the largest changes with respect to interaction robustness with several “hidden” non-adjacent pairs. We also computed the differential co-expression (see Methods) for case of breast cancer (both BRCA^M^ and BRCA^T^) and refer the readers to previous work for computational details^20^. In particular, we observed the ranking of interactions of differential co-expression to that of differential Ollivier-Ricci curvature vastly differ (Fig. S2), i.e., we are uncovering hidden information of the underlying system. Similar observations were gleaned from the remaining sets; however, we focus on breast cancer for the sake of brevity.

To this end, we also applied our method to analyze metabolic genes for the case of breast cancer, i.e., BRCA^M^ (Table S4). While the data did not include various associated cancer genes (i.e., TP53, KRAS, BRAF), we were able to uncover several lesser known targets. In particular, we observed at the top of our list, the gene LPO which has been known to contribute to the initiation of breast cancer^27^, SOD3 has been considered an important gene in the defense against oxidative stress and prevention of estrogen-mediated breast cancer^28^, GOT2 has been noted to significantly affect cell growth^29^, and over-expression of LRAT has lead to a poor prognoses in colorectal cancer^30^. While a complete analysis in the context of cancer biology will be a subject of future work, the above results should be placed in the context “lost information” due to the resolution limitations of network entropy (see Discussion). In short, we now have a proxy for robustness at the local interaction level.

### Gene Robustness: Scalar Curvature

Until now, we have considered Ricci curvature (in the Ollivier sense), which is defined between *any* two vertices on a graph. This is the main focus of the present work. However, in order to compare the curvature based approach with that of network entropy^4^, we now define several nodal measures based on the notion of “scalar curvature.”

In standard geometry, scalar curvature represents the amount by which the volume of a geodesic ball in a curved Riemannian manifold deviates from that of the standard ball in Euclidean space^8^. On a weighted graph, it may be defined in an analogous manner as:


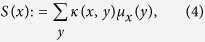


where we contract Ollivier-Ricci curvature with respect to measure *μ*_*x*_(*y*). Analyzing this contraction, we note that the measure *μ*_*x*_(*y*) serves as a normalization factor that attempts to remove biasing with regards to topology (i.e., node degree). We can analogously define the unnormalized scalar curvature by contracting with respect to the hop metric, i.e.,


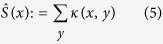


where the summation runs over all *y* such that *d*(*x*, *y*) = 1.

One may also consider measures where nodal curvature at *x* in its adjacent neighborhood can be defined as its minimum (maximum) Ollivier-Ricci curvature. Given that lower bounds of Ricci curvature are connected to entropy^9^, attaching this bound as a measure yields yet another characterization of nodal robustness. We should note that contracting with respect to the measure *μ*_*x*_(*y*) is in the spirit of local normalized entropy defined in previous cancer studies to be 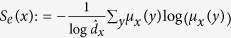
4. Similarly, contracting with respect to hop metric above is very much in line with the unnormalized entropy, i.e., 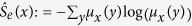
7.

After evaluating the above measures on all cancer networks for which we had data, we found that the results are consistent and comparable in the sense of cancer network differentiation and present an average nodal measure for each cancer study along with the p-value of a one-tailed paired Wilcoxon signed-rank test (Table 4, Table S5, Table S6). We see that there exists a positive shift in the distribution for both entropy and curvature with the exception of only one case where the p-value for Δ*S* in HNSC^T^ was insufficient (Table S6). Given that our primary focus of this work is on the interaction level, we present the top and bottom ten pairs in the BRCA^T^ network with respect to normalized scalar curvature (Table 5, Table S7). This is done, in part, to illustrate the unavoidable “information loss” of any nodal measure chosen. For example, we observe that although the some genes (i.e., RNF43, ETV1) possess the strongest robust interactions, they are listed in the bottom list with respect to scalar curvature. As we will argue in the next section, emphasis should be placed on interactions when analyzing network robustness.

## Discussion

In this work, we have presented a framework to quantify interaction gene-to-gene robustness through the notion of Ollivier-Ricci curvature with an application to cancer networks. This was motivated through the intrinsic connection between entropy and Ricci curvature, and in turn, robustness via the Fluctuation Theorem. From this, we demonstrated that cancer tissue exhibits a higher curvature at the interaction and gene level on all the networks tested. While these two measures may provide important biological information^4^, it is important to first discuss the differences in the context of our findings and in general, cancer biology. As the eventual goal is to uncover “knock-down” targets (and the effect thereof), we must also explore how one can alter network properties with respect to robustness including changes to the network geometry and topology.

We begin by revisiting the top and bottom gene-to-gene interactions of breast cancer in the studied network composed of known cancer-related genes. At the *interaction* level, changes in robustness need not be restricted to simply a negative/positive change–genes will tend to interact in a wide ranging manner and may contain seemingly important interactions not explicitly defined by the underlying geometry and topology. From equation (4), we clearly see that through the contraction, we “lose” information through a (weighted) average in two distinct manners. Firstly, RNF43 possesses two of the strongest and weakest pairs–averaging these together will cancel out their relative significance. Secondly, nodal measures take an average over an adjacent neighborhood thereby ignoring those interactions that are non-adjacent. As we can see, several important interacting pathways (e.g., RNF43-POT1) should not be ignored as these gene-to-gene interactions exhibit larger changes than many interactions that are adjacent. The same arguments hold for network entropy. Further, previous work on network entropy discusses the significance and careful attention one must have with respect to the topology biasing. Hence, normalization factors are often adopted to provide insight into the nodal robustness^4^,^7^. No such normalization is required when employing Ollivier-Ricci curvature.

Further, the development of a systematic approach to altering network properties to uncover potential drug targets is key. In particular, certain targets may not be directly “druggable” thereby requiring one to alter a set of genes/interactions that provide similar impact. That is, simply choosing a “knock-down” gene on nodal robustness may prove to be insufficient. To this end, one may consider the corresponding Ricci flow:


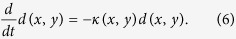


Not much is known about this flow, but the idea would be, while keeping the same topology, one would change the graph weights, or the network of links among the nodes, in such a way as to uniformize the curvature *κ*. In the engineering literature^31^, this has been offered as an approach, in the case of certain wireless networks, to have the effect of removing some of the overloaded queues, and thus should have important implications for cancer networks. Understanding discrete analogues of Ricci flow in this connection will be considered as a future research topic in this connection.

Next, we would like to mention some very interesting work^32^ that describes a metric geometry on the space of trees in connection with phylogenetics. It turns out that their space is a moduli space (universal parameter space) and has non-positive curvature. From previous results^33^, this allows one to do statistics on this space since between any two points there is a unique geodesic. This has had a number of intriguing applications in cancer research^34^. It would be very interesting to generalize this to more general network structures, and instead of just looking at the geometric (curvature) property of an individual network to devise quantitative statistical methods based on the metric geometry comparing families of networks.

Finally, the work of Rabadan^34^ has been largely motivated by the problem of cancer cell heterogeneity. Indeed, cancer progression is believed to follow Darwinian evolutionary pattern: fitter subtypes replace other less fit cells, which leads to disease. In combination with high-throughput genomics one can construct trees to study this process. This is an example of a deep relationship between the concepts of Darwinian evolution and Boltzmann thermodynamics^6^. The idea is that macroscopic entropy increases under microscopic molecular collisions, while macroscopic evolution can be (partially) explained via the concept of the increase of entropy. This reasoning is very much in line with the overall thrust of the present paper in which we are trying to use curvature (positively correlated with robustness) to quantify network robustness. The macroscopic theory is very much in line with Boltzmann thermodynamics. Evolutionary changes and network adaptability are key topics to be considered in future research.

## Methods

### Data

All TCGA expression data were accessed using the Broad Institute Firehose on November 4, 2014.

Two distinct approaches were used to determine adjacency matrices for the networks under study. For our study of networks of cancer-related genes from Cosmic^21^, we used the simple interaction data provided by Pathway Commons project (v6 - accessed in February, 2015 from http://www.pathwaycommons.org/pc2/downloads). To do this, we first downloaded the binary relationships between pairs of genes in Simple Interaction Format. We then filtered the data only for interaction type “neighborhood-of” that represents any type of pathway-based interaction between a pair of genes. We next filtered out all interactions in which either of the interacting genes was not in our cancer gene set, and therefore not of interest to us.

To identify adjacent edges in the metabolic gene data set, we used the Recon2 human metabolic reconstruction^22^ to identify pairs of genes whose enzymatic products shared a common substrate or product. To do so, we pruned the stoichiometric matrix (*S*) for cofactors (ATP, ADP, NADH, NAD, NADPH, NADP, etc.) and other highly-connected metabolites which might adversely affect the adjacency calculations (*e.g.* water, hydrogen ions, metal cofactors). We then used this pruned stoichiometric matrix *S*_*P*_, and the reaction-to-gene matrix (*R*) to generate a matrix encoding which metabolites and genes participated in common reactions (*MG* = *S* × *R*). Finally, to generate an adjacency matrix (*A*) indicating which genes participated in reactions sharing a common metabolite, we multipled the transpose of *MG* by itself: *A* = *MG*^*T*^ × *MG*. The matrix *A* is square, with the length of each dimension equal to the number of genes in the model. The curvature analysis was also repeated after removing highly connected reactions (*i.e.* with greater than 4 distinct metabolite substrates/products, after pruning for highly connected metabolites) from *S* with qualitatively similar results.

For each data set, gene co-expression networks were generated by calculating the non-parametric (Spearman) correlation between all pairs of genes. Note that since we are working with correlation data for which values can be less than zero, the analysis was conducted with respect to the transformed correlation coefficient: 
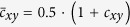
 in order to construct the random walk over the network^4^. We should also note, that one could examine and compute weights through an interesting mass action approach^13^,^14^ as opposed to a more general computation of correlation values given above. The advantage of the mass action method is that from an statistical standpoint, it allows for the analysis to be carried out in a more sample specific manner. Moreover, given that biological networks involve both negative and positive weights representing specific activating and inhibiting interactions, a subject of future research will entail directly extending the current approach to more general directed graph case following related work^35^.

### The Wasserstein Distance

We begin by recording the basic definition of the *L*^*p*^-Wasserstein distance from optimal transport theory that we will need below. Roughly speaking, on a metric measure space, one gets a natural distance on “small” balls around points or the “fuzzified” points. For full details about the Monge-Kantorovich (optimal mass transport) problem and the associated Wasserstein distance, we refer the reader to several works on this topic^23^,^24^,^36^,^37^,^38^.

More precisely, let *X* be a metric measure space, equipped with distance *d*. Let *μ*_*i*_, *i* = 1, 2, be two measures with the same total mass and finite *p*-th moment. A *coupling* between *μ*_1_ and *μ*_2_ is a measure *μ* on *X* × *X* such that


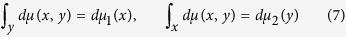


In other words, the marginals of *μ* are *μ*_1_ and *μ*_2_. Let Π(*μ*_1_, *μ*_2_) be the set of couplings between *μ*_1_ and *μ*_2_. We then define the *L*^*p*^ *Wasserstein distance* as


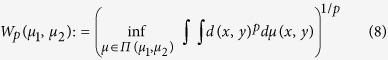


In this paper, we only consider the cases *p* = 1, 2. For *p* = 1, the Wasserstein distance is sometimes called the “Kantorovich-Rubinstein distance” or the Earth Mover’s distance (EMD) and can be formulated as linear program^12^. In particular, let *X* denote a discrete metric measure space with *n* points denoted {*x*_1_,…,*x*_*n*_}. Let *μ*_1_ and *μ*_2_ be two distributions, and let *d*(*x*, *y*) denote the distance between *x*, *y* ∈ *X* (for the case of graphs, this is simply taken to be the hop distance). We assume that *μ*_1_ and *μ*_2_ have the same total mass. Then, *W*_1_(*μ*_1_, *μ*_2_) may be defined as follows:


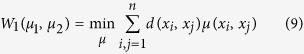


where 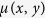 is a coupling (or flow) subject to the following constraints:


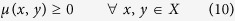



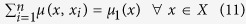



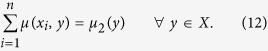


The cost above finds the optimal coupling of moving a set of mass from distributions *μ*_1_ to *μ*_2_ with minimal “work”.

### Curvature and Robustness

There have been a number of approaches^19^,^39^,^40^,^41^ to extending the notion of Ricci curvature to more general metric measure spaces. At this point, the exact relationship of one approach as compared to another is unclear. Roughly, the techniques fall into two categories: the first generalizing the weak *k*-convexity of the entropy functional on the Wasserstein space of probability measures as in^9^,^39^,^42^, and the second directly working with Markov chains to define the generalization^19^,^40^,^41^ on networks. There is also a notion of “hyperbolicity” due to Gromov^43^ based on the “thinness” or “fatness” of triangles compared to the Euclidean case, and more generally a certain four-point criterion. Depending upon the application, each approach seems to be useful. In particular, we follow^19^,^39^, because of connections to notions of metric entropy.

We first define the precise notion of “robustness” to which the Fluctuation Theorem^6^,^44^, is applicable. One considers random fluctuations (perturbations) of a given network that result in deviations of some observable. Let *P*_*ε*_(*t*) denote the probability that the mean deviates by more than *ε* from the original (unperturbed) value at time *t*. Since *P*_*ε*_(*t*)→0, we want to measure its relative rate, that is, we set


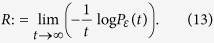


Therefore, large *R* means not much deviation and small *R* large deviations. In thermodynamics, it is well-known that entropy and rate functions from large deviations are very closely related.

Next we describe the relationship of curvature and entropy given in Lott and Villani^9^. Let (*X*, *d*, *m*) denote a geodesic space, and set


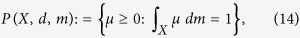



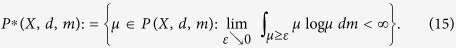


We define


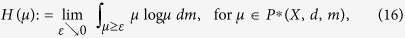


which is the negative of the *Boltzmann entropy S*_*e*_(*μ*): = −*H*(*μ*); note that the concavity of *S*_*e*_ is equivalent to the convexity of *H*. Then we say that *X* has *Ricci curvature bounded from below by k* if for every *μ*_0_, *μ*_1_ ∈ *P*(*X*) there exists a constant speed geodesic *μ*_*t*_ with respect to the Wasserstein 2-metric connecting *μ*_0_ and *μ*_1_ such that


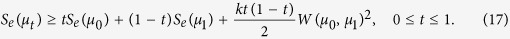


This indicates that entropy and curvature are *positively correlated* that we will express as


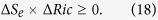


We note here that changes in *robustness*, i.e., the ability of a system to functionally adapt to changes in the environment (denoted as Δ*R*) is also positively correlated with entropy via the Fluctuation Theorem^6^,^44^, and thus with network curvature:


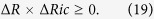


Since the curvature is very easy to compute for a network, this may be used as an alternative way of expressing functional robustness. This being said, we adopt the recent notion of Ollivier-Ricci curvature motivated from coarse geometry^18^,^19^.

### Ornstein-Uhlenbeck Process

It is very informative to consider the relationship of the the Ollivier-Ricci curvature and robustness via a simple example^18^,^19^. We consider the Ornstein-Uhlenbeck process. The latter is a modification of the Wiener process (random walk), in which there is a tendency to converge to a central location.

More precisely, consider the stochastic differential equation


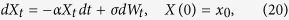


where *W* is Brownian motion (Wiener process), and we take *x*_0_ to be deterministic. We treat the 1-dimensional case for simplicity. Everything goes through in higher dimensions as well. The corresponding Fokker-Planck equation is


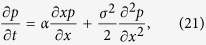


where *p* = *p*(*x*, *t*|*x*_0_, 0) is the transition probability of the underlying Markov process. One may show that *p*(*x*, *t*|*x*_0_, 0) is a Gaussian process with mean and variance given by^45^:


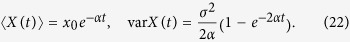


We see that we get transition probabilities of mean *x*_0_*e*^−*αt*^ and variance independent of *x*_0_. Since all the transitions *p*(*x*, *t*|*x*_0_, 0) have the same variance (and are Gaussian) the 1-Wasserstein distance^46^


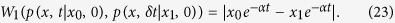


Finally,


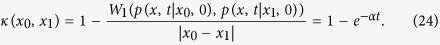


Equation (24) illustrates the connection of fluctuation in a very simple explicit manner. Larger *α* corresponds to larger curvature *κ* and this corresponds to how quickly the systems returns to equilibrium, that is to the mean going to 0.

### Convergence to Invariant Distribution

One can also see that relationship of robustness to the Ollivier-Ricci curvature in the following manner^18^ dealing with Markov chains. The basic idea is that larger Ollivier-Ricci curvature indicates greater robustness via rate of convergence to the invariant (equilibrium) distribution. Specifically, suppose *κ*(*x*, *y*) ≥ *k* > 0. Then there exists a unique invariant probability measure *v*. Moreover, for any *x*,


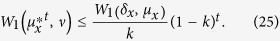


Here,


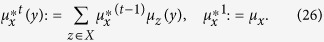


Note that *W*_1_(*δ*_*x*_, *μ*_*x*_) represents the jump of the random walk at *x*. On a connected graph *X* with diameter *D* (defined as the longest graph geodesic), this yields the following estimate for the mixing time:


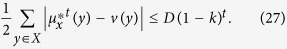


This example combined with the previous one provides further support that Ollivier-Ricci curvature can be employed as a natural proxy for robustness with the the distinct advantage of being easily computable.

### Differential (Co-)Expression

We conclude with a simple computation of differential co-expression. Following previous work^20^, differential co-expression was computed using the (non-transformed) sample correlation coefficient *c*_*xy*_ by first applying the Fisher *z*-transformation in order to stabilize variances due to population size:


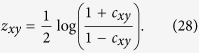


If we let 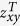 and 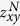 denote the *z*-transformation for cancer and normal gene pairs, respectively, one can then compute the differential co-expression as


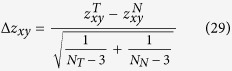


where *N*_*T*_ and *N*_*N*_ is the number of tumor and normal samples respectively. For differential expression values, we summed those co-differential values for a given gene’s interaction defined by the underlying adjacency matrix. This was done in order to provide a fair comparison to the values computed by scalar curvature. We again note that complete information regarding this method and data can be found in previous work^20^.

## Additional Information

**How to cite this article**: Sandhu, R. *et al.* Graph Curvature for Differentiating Cancer Networks. *Sci. Rep.* **5**, 12323; doi: 10.1038/srep12323 (2015).

## Supplementary Material

Supplementary Information

## Figures and Tables

**Figure 1:**
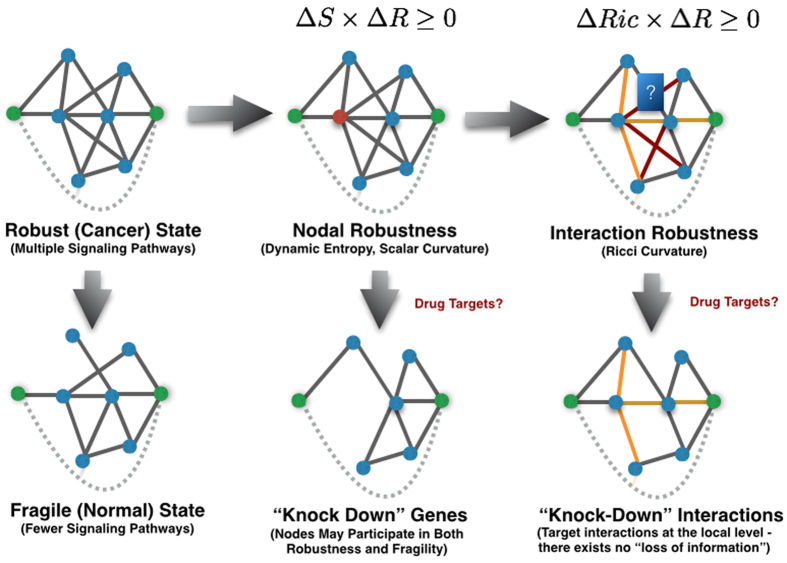
This work focuses on analyzing robustness with respect to pairwise interactions. Systems equipped with multiple signaling pathways can be framed in the context of robustness. Whereas previous work has shown dynamic entropy as a cancer “hallmark” through a nodal characterization, we expound upon this by providing a framework to analyzing gene-to-gene interaction robustness. In doing so, we will show that the method herein presents no “loss of information” and may be aptly suited to uncover particular pathways contributing to the robustness of cancer systems.

**Table 1:** A distribution analysis for changes in Ollivier-Ricci average curvature between cancer and normal tissue for all metabolic case studies.

Ricci Curvature	BRCA^M^	HNSC^M^	KIRP^M^	LIHC^M^	LUAD^M^	THCA^M^
Δ Average	0.0195	0.0186	0.0229	0.0035	0.0075	0.0117
p-Value	<1e-25	<1e-25	<1e-25	<1e-25	<1e-25	<1e-25
Δ 5% Left Tail	0.0059	0.0077	0.0077	0.0016	0.0022	0.0013
Δ 5% Right Tail	0.0059	0.0057	0.0059	0.0005	0.0017	0.0010

These statistics show that there exists a positive shift in the distribution signifying robustness. We also include the one tailed pair Wilcoxon signed rank test p-values to support the above statistics.

**Table 2:** Top 10 pairs with respect to changes in Ollivier-Ricci curvature in BRCA^*T*^.

Gene Ranking	∆ Ricci Curvature (Cancer-Normal)	Differential Co-Expression	Gene X (Symbol)	Gene Y (Symbol)
1	0.3504	2.7316	RNF43	RSPO3
2	0.3444	2.2061	RNF43	RSPO2
3	0.3012	11.6989	ERG	ETV1
4	0.3001	1.1143	GPC3	PTCH1
5	0.2901	6.6544	GPC3	SDC4
6	0.2796	5.3742	POT1	SBDS
7	0.2538	5.1751	FGFR2	KDR
8	0.2509	−1.1166	ETV1	FEV
9	0.2460	5.2252	ERG	FOXA1
10	0.2410	3.0769	EXT1	SDC4

**Table 3:** Bottom 10 pairs with respect to changes in Ollivier-Ricci curvature in BRCA^*T*^.

Gene Ranking	∆ Ricci Curvature (Cancer-Normal)	Differential Co-Expression	Gene X (Symbol)	Gene Y (Symbol)
99226	−0.2203	−6.2393	POT1	RNF43
99227	−0.2214	1.8210	ELF4	FEV
99228	−0.2304	−7.0964	MUC1	RNF43
99229	−0.2489	−3.5280	DH1	SBDS
99230	−0.2496	−5.7021	ERCC2	RNF43
99231	−0.2839	−3.6199	PRDM1	RNF43
99232	−0.2851	−2.3147	CCND3	ETV1
99233	−0.3632	−6.8679	RNF43	SFPQ
99234	−0.3636	−8.0784	NONO	RNF43
99235	−0.3651	−8.6880	RNF43	TP53

**Table 4:** Comparison of different nodal measures for curvature and entropy on all networks composed of metabolic genes.

Measure	BRCA^M^	HNSC^M^	KIRP^M^	LIHC^M^	LUAD^M^	THCA^M^
Δ S	0.0119	0.8311	0.0139	0.0036	0.0036	0.0026
p-Value	<1e-25	<1e-25	<1e-25	<7e-14	<4e-21	2e-7
Δ Ŝ	0.8311	0.7925	0.9737	0.1483	0.3205	0.1365
p-Value	<1e-25	<1e-25	<1e-25	<1e-25	<1e-25	<1e-25
Δ S_*e*_	0.0116	0.0113	0.0144	0.0025	0.0047	0.0027
p-Value	<1e-25	<1e-25	<1e-25	<1e-25	<1e-25	<1e-25
Δ Ŝ_*e*_	0.0373	0.0353	0.0474	0.0070	0.0147	0.0077
p-Value	<1e-25	<1e-25	<1e-25	<1e-25	<1e-25	<1e-25

**Table 5:** Bottom 10 genes in BRCA^*T*^ ranked with respect to scalar curvature.

Gene Ranking	∆ Scalar Curvature (Cancer-Normal)	Differential Expression	Gene (Symbol)
457	−0.0761	−8.5248	SDHD
458	−0.0792	1.8463	SBDS
459	−0.0797	−18.6966	RNF43
460	−0.0841	1.4997	FLI1
461	−0.0884	−3.5989	MYCN
462	−0.1213	6.2235	ETV1
463	−0.1268	2.7942	SDC4
464	−0.1313	4.2820	TFEB
465	−0.1478	1.0500	ELN
466	−0.1870	10.0541	FEV
