# Supplementary material for: Graph Curvature for Differentiating Cancer Networks
Source: Sci Rep. 2015 Jul 14;5:12323. doi: 10.1038/srep12323 (PMC4500997; doi:10.1038/srep12323)
Supplement: Supplementary Information [file srep12323-s1.pdf]

SUPPLEMENTARY INFORMATION FILE

Graph Curvature for Differentiating Cancer Networks

Romeil Sandhu[1+], Tryphon Georgiou[2], Ed Reznik[2], Liangjia Zhu, Ivan Kolesov[1], Yasin Senbabaoglu[2], and Allen Tannenbaum[1+*]

[1] Departments of Computer Science and Applied Mathematics/Statistics, Stony Brook University, Stony Brook, NY 11794

[2] Department of Electrical and Computer Engineering, University of Minnesota, Minneapolis, MN 55455

[3] Computational Biology, Memorial Sloan Kettering, New York, NY 10065

+These authors contributed equally to this work

*corresponding author: allen.tannenbaum@stonybrook.edu

SUPPLEMENTARY FIGURES:

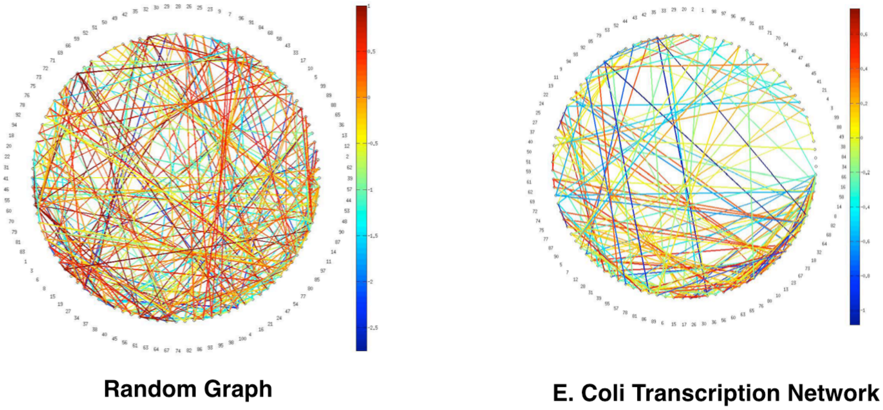

Supp. Fig. 1: We computed the Ollivier-Ricci curvature of a random network and of a transcription network of E. Coli: darker colors represent more negative curvature. The average Ollivier-Ricci curvature for the random network and E. Coli was -0.3520 and -0.0222, respectively. This supports previous evidence regarding inherent biological robustness of such networks exhibiting motifs to that of a random network.

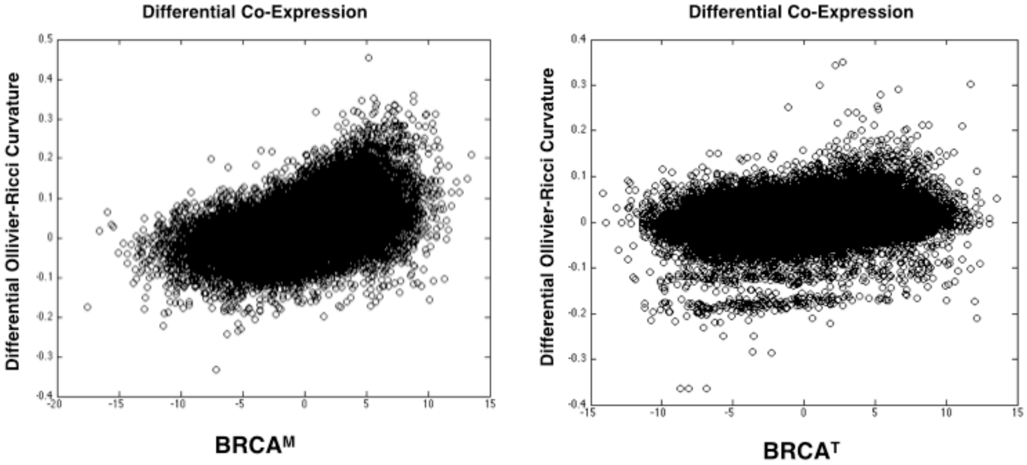

Supp. Fig. 2: We computed and plot the differential co-expression with that of differential Ollivier-Ricci curvature to illustrate the uncovering of new information from the underlying network. In particular, the ranking of gene-to-gene interactions are different and the above plot is presented for the case of breast cancer. Similar results hold for remaining networks studied in the present work.

SUPPLEMENTARY TABLES:

Nodal Measure	$BRCA^T$	$HNSC^T$	$LUAD^T$	$THCA^T$	$LIHC^T$	$KIRP^T$
ΔAverage p-value	0.0117 <1e-25	0.0039 <1e-25	0.0127 <1e-25	0.0059 <1e-25	0.0083 <1e-25	0.0095 <1e-25
Δ5% Left Tail Average	0.0112	0.0028	0.0077	0.0037	0.0101	0.0047
Δ5% Right Tail Average	0.0038	0.0005	0.0068	0.0023	0.0043	0.0038

Supp. Table 1: A distribution analysis for changes in Ollivier-Ricci average curvature between cancer and normal tissue for all case studies involving a subset of known cancer related genes (denoted by ^{T}). These statistics show that there exists a positive shit in the distribution signifying robustness. We also include the one tailed pair Wilcoxon signed rank test p-values to support the above statistics

Left Tail Length (%)	$BRCA^M$	$HNSC^M$	$KIRP^M$	$LIHC^M$	$LUAD^M$	$THCA^M$
0.10%	0.0160	0.0117	0.0145	0.0041	0.0085	0.0025
0.50%	0.0111	0.0115	0.0121	0.0036	0.0052	0.0023
1.00%	0.0097	0.0107	0.0117	0.0034	0.0035	0.0021
3.00%	0.0072	0.0086	0.0095	0.0018	0.0024	0.0014

Supp. Table 2: This table provides the difference in expected value of varying tail lengths for all network composed of metabolic genes conducted in this paper.

Left Tail Length (%)	$BRCA^T$	$HNSC^T$	$LUAD^T$	$THCA^T$	$LIHC^T$	$KIRP^T$
0.10%	0.0359	0.0186	0.0359	0.0006	0.0158	0.0198
0.50%	0.0185	0.0100	0.0104	0.0006	0.0122	0.0047
1.00%	0.0128	0.0072	0.0078	0.0036	0.0138	0.0074
3.00%	0.0087	0.0033	0.0056	0.0047	0.0123	0.0069

Supp. Table 3: This table provides the difference in expected value of varying tail lengths for networks composed of known cancer related genes conducted in this paper.

Gene Rank	Δ Ollivier-Ricci Curvature (Cancer-Normal)	Gene X (Symbol)	Gene Y (Symbol)
1	0.4642	ACAA2	MYO5B
2	0.4170	LPO	SOD3
3	0.4162	BPNT1	GLYCTK
4	0.4067	GLYCTK	GAL3ST1
5	0.4057	PPAP2A	PPAP2C
6	0.3916	ACO1	OAT
7	0.3881	HADHB	MYO5B
8	0.3805	GUCY2D	NPR2
9	0.3687	GUCY2D	NPR1
10	0.3624	GOT2	ACO1

Supp. Table 4: This table provides the top 10 gene interactions with respect to Ollivier- Ricci curvature for the case of $BRCA^M$.

Nodal Measure	$BRCA^M$	$HNSC^M$	$KIRP^M$	$LIHC^M$	$LUAD^M$	$THCA^M$
ΔS_{min} p-value	0.0150 <1e-25	0.0118 8e-16	0.0121 4e-11	0.0057 7.5e-7	0.0070 9e-8	0.0044 2e-4
ΔS_{max} p-value	0.0344 <1e-25	0.0366 <1e-25	0.0341 <1e-25	0.0102 <1e-25	0.0203 <1e-25	0.0109 7e-23

Supp. Table 5: We provide two additional nodal measures for which we attach the minimum (and maximum) Ollivier Ricci Curvature at node x in an adjacent neighborhood of y for the network composed of metabolic genes. Similar to that of nodal measures discussed in the present work, we take the average over nodal measures. One can see that all networks exhibit, on average, higher nodal robustness with respect to these measures as well. We also provide p-values for one-tailed paired Wilcoxon signed rank test.

Nodal Measure	BRCA^{T}	HNSC^{T}	LUAD^{T}	THCA^{T}	LIHC^{T}	KIRP^{T}
ΔS	0.0024	0.0015	0.0048	0.0048	0.0056	0.0035
p-value	2.5e-5	4e-1	2e-8	1e-6	5e-15	7e-3
$\Delta\hat{S}$	0.4292	0.1423	0.4638	0.2177	0.3025	0.3471
p-value	2e-16	2e-5	5e-20	5e-11	7e-23	4e-13
$\Delta S_{\min}$	0.0157	0.0121	0.0116	0.0030	0.0076	0.0086
p-value	3e-3	7e-7	7e-6	3e-2	2e-5	5e-5
$\Delta S_{\max}$	0.0123	0.0143	0.0121	0.0052	0.0105	0.0124
p-value	9e-6	1e-7	4e-11	7e-4	1e-7	2e-8
ΔS_e	0.0117	0.0079	0.0079	0.0037	0.0042	0.0075
p-value	<1e-25	<1e-25	<1e-25	<1e-25	<1e-25	<1e-25
$\Delta\hat{S}_e$	0.0303	0.0213	0.0221	0.0102	0.01116	0.0202
p-value	<1e-25	<1e-25	<1e-25	<1e-25	<1e-25	<1e-25

Supp. Table 6: We provide nodal measures for networks involving a subset of known cancer related genes (denoted by ^{T}) along with the one tailed paired Wilcoxon signed rank test.

Gene Rank	Δ Scalar Curvature (Cancer-Normal)	Differential Co-expression	Gene (Symbol)
1	0.1477	1.8524	RSPO2
2	0.1415	2.3779	RSPO3
3	0.0931	227.8612	PICALM
4	0.0910	91.1372	SDHB
5	0.0707	57.1017	KIT
6	0.0646	35.9899	CEBPA
7	0.0645	2.6437	PDRM1
8	0.0582	142.9690	CLTCL1
9	0.0561	44.3039	TAL1
10	0.0554	116.3448	CLTC

Supp. Table 7: This table provides the top 10 genes with respect to scalar curvature for the case of BRCA^{T}.
